# Hemorrhagic polyps formed like fundic gland polyps during long-term proton pump inhibitor administration

**DOI:** 10.1007/s12328-017-0756-x

**Published:** 2017-06-28

**Authors:** Tsutomu Takeda, Daisuke Asaoka, Yuzuru Tajima, Kenshi Matsumoto, Naoto Takeda, Takahumi Hiromoto, Shoki Okubo, Hiroaki Saito, Tomonori Aoyama, Tomoyoshi Shibuya, Naoto Sakamoto, Mariko Hojo, Taro Osada, Akihito Nagahara, Takashi Yao, Sumio Watanabe

**Affiliations:** 1 Department of Gastroenterology, Juntendo University School of Medicine, Tokyo, Japan; 2 Department of Human Pathology, Juntendo University School of Medicine, Tokyo, Japan; 3 Department of General Medicine, Juntendo University School of Medicine, Tokyo, Japan

**Keywords:** Fundic gland polyp, Long-term proton pump inhibitor therapy, Bleeding, Selective serotonin reuptake inhibitors, Aquaporin-4, KCNQ1

## Abstract

We report a rare case of hemorrhagic gastric polyps resulting in anemia during long-term proton pump inhibitor (PPI) administration that endoscopically looked like a fundic gland polyp (FGP). A 44-year-old man presented complaining of anemia and tarry stools. Esophagogastroduodenoscopy (EGD) demonstrated multiple white edematous polyps in the corpus and antrum, which were considered to be FGPs. We attempted endoscopic hemostasis but hemorrhaging increased because of hemorrhagic polyps and vulnerable gastric mucosa. Re-bleeding occurred several times. Polyp resection was performed at 24 polyp sites. We also ceased the administration of PPI. Microscopically, polyps showed characteristics of hyperplasia in the foveolar epithelium, extensions of fundic glands, and edema of the stroma. The proliferation of parietal and chief cells was also observed. Immunohistochemically, aquaporin-4 (AQP4) and KCNQ1-positive parietal cells and dilated mucous glands were found from the basal side to the apical side of the mucosa. These findings were compatible with the development of lesions associated with the long-term administration of PPI. EGD revealed an improvement in the vulnerability of gastric mucosa and the development of polyps, with no further gastric polyps observed 1 year after discharge. Bleeding from polyps resembling FGPs is generally rare, with indications that long-term PPI administration may induce such bleeding.

## Introduction

Proton pump inhibitors (PPI) are important drugs used worldwide as first-line drugs for gastroesophageal reflux disease and non-steroidal anti-inflammatory drug-induced ulcer treatment. However, in recent years, the increased risk of gastric polyps during long-term PPI administration has been a growing concern. In this case report, we describe rare hemorrhagic gastric polyps, resulting in anemia during long-term PPI administration, which formed into fundic gland polyps (FGPs) as determined endoscopically, but which were not typical for FGP as determined by pathology.

## Case report

A 44-year-old man presented to hospital complaining of anemia and tarry stools. He had previously reported heartburn symptoms 10 years previously. Since a subsequent esophagogastroduodenoscopy (EGD) did not show any abnormalities, he was diagnosed with non-erosive reflux esophagitis and the oral administration of 15 mg lansoprazole was initiated once a day. He also had a history of hypertension and depression, therefore paroxetine hydrochloride hydrate (20 mg/day for 7 years), and amlodipine besilate (10 mg/day) had been administered for several years.

**Fig. 1:**
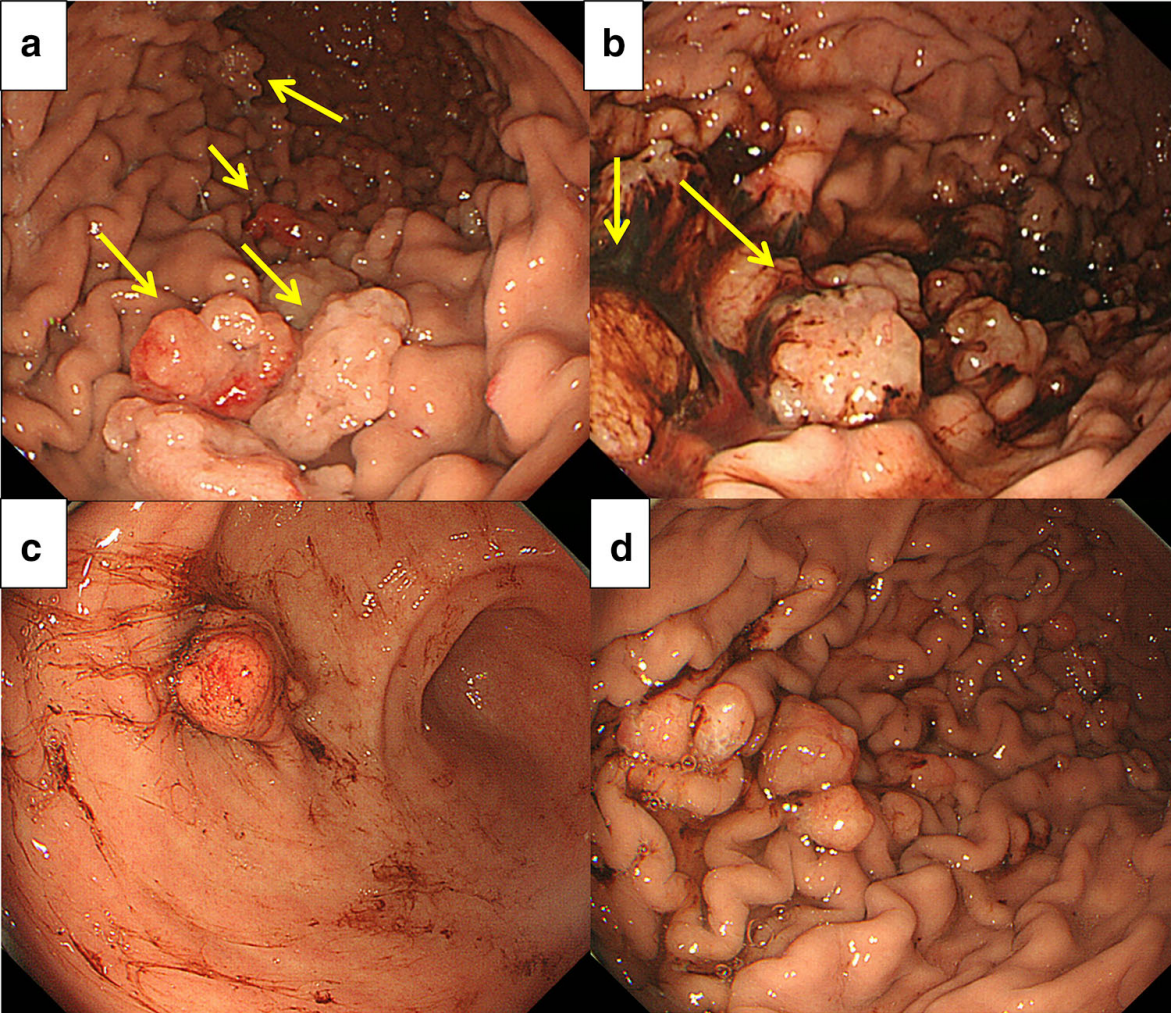
Esophagogastroduodenoscopic findings. **a** Multiple white edematous polyps (*arrows*) were observed in the corpus and antrum, which were considered to be fundic gland polyps (FGPs) as determined endoscopically before admission. **b** Esophagogastroduodenoscopy (EGD) revealed mild oozing from polyps of the gastric corpus (*arrows*) after admission. **c** EGD findings at the time of readmission: a reddish, hemorrhagic polyp was observed in the antrum, together with coagula. **d** Coagula were observed in the stomach, and multiple polyps in the corpus were hemorrhagic

**Fig. 2:**
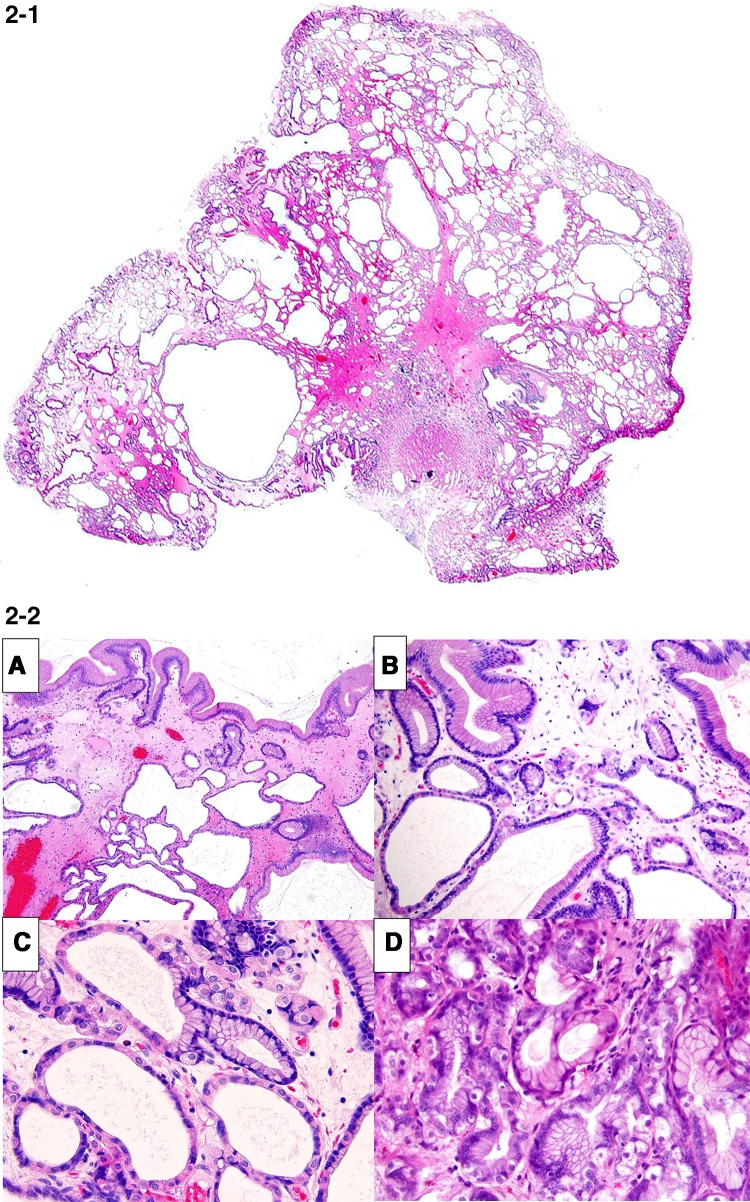

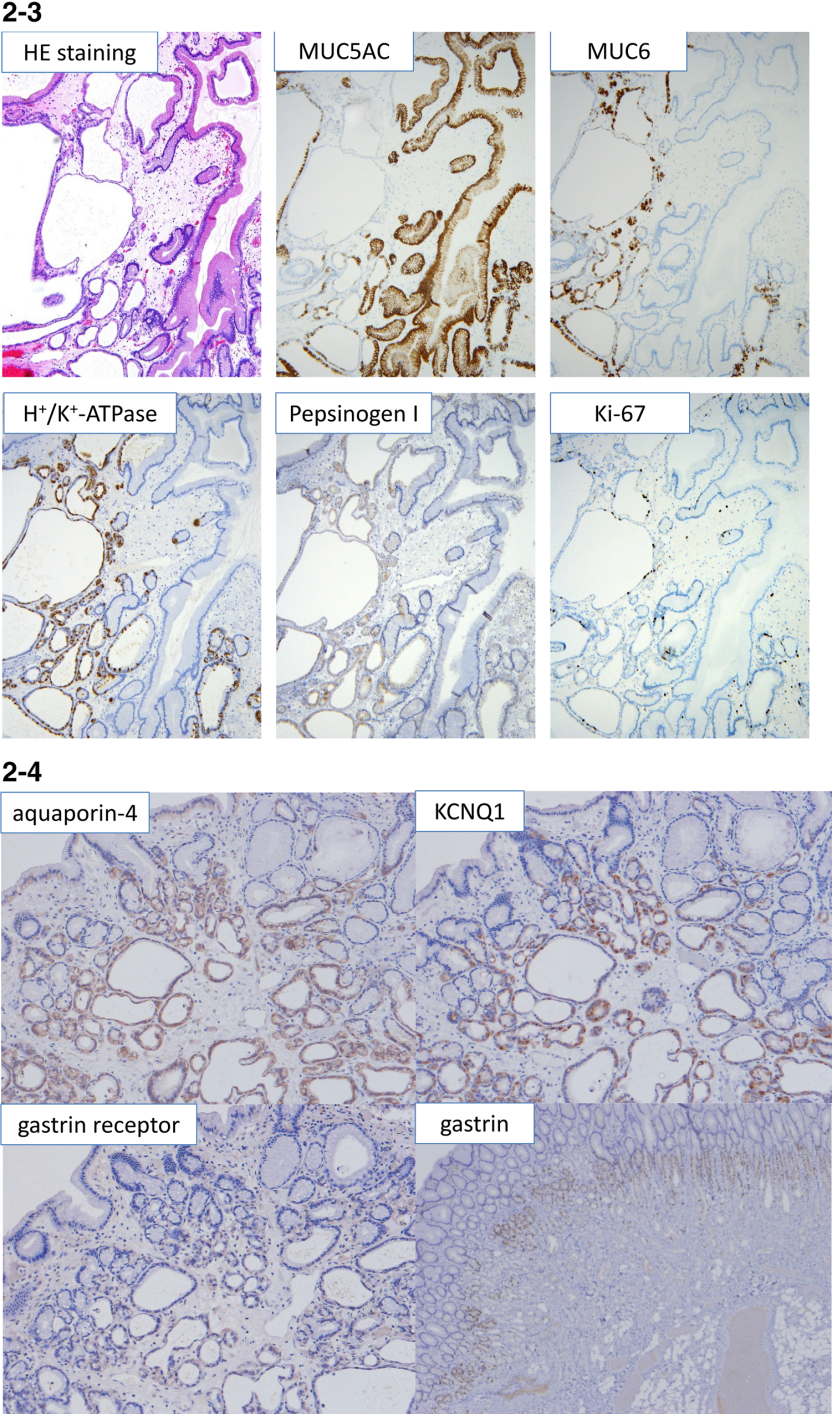
**1** Pathological findings of resected polyps that looked like fundic gland polyp (hematoxylin & eosin [HE] staining, loupe image). Polyps showed characteristics of hyperplasia of the foveolar epithelium, extended fundic glands and edema of the stroma. **2** Characteristics of pathological findings of resected polyps that looked like fundic gland polyp (hematoxylin & eosin [HE] staining). Mixed with hyperplasia of foveolar epithelium and extended mucous glands (*A*), the proliferation of parietal and chief cells was also observed (*B*), but not parietal cell protrusion or inflammatory cell infiltration (*C*). Apoptotic bodies were detected in the boundary region between fundic and neck mucous glands (*D*). **3** Immunohistochemistry for MUC5AC, MUC6, H^+^/K^+^-ATPase, pepsinogen I, Ki-67. Crypt epithelial cells (MUC5AC) were mainly observed superficially, and cervical mucous cells (MUC6) were observed beneath the crypt epithelium. Fundic glands were positive for H+/K+-ATPase, and showed proliferation of predominantly parietal cells. Few Ki67 positive cells were found. **4** Immunohistochemistry for aquaporin-4, KCNQ1, gastrin and gastrin receptor. Aquaporin-4 (AQP4) and KCNQ1-positive parietal cells and dilated mucous glands were found from the basal side to the apical side of the mucosa. The extension of the distribution of AQP4 and KCNQ1-positive cells toward the apical side of the fundic glands were observed. Overexpression of gastrin receptors was not detected. Gastrin overexpression was not observed in the antral polyp

**Fig. 3:**
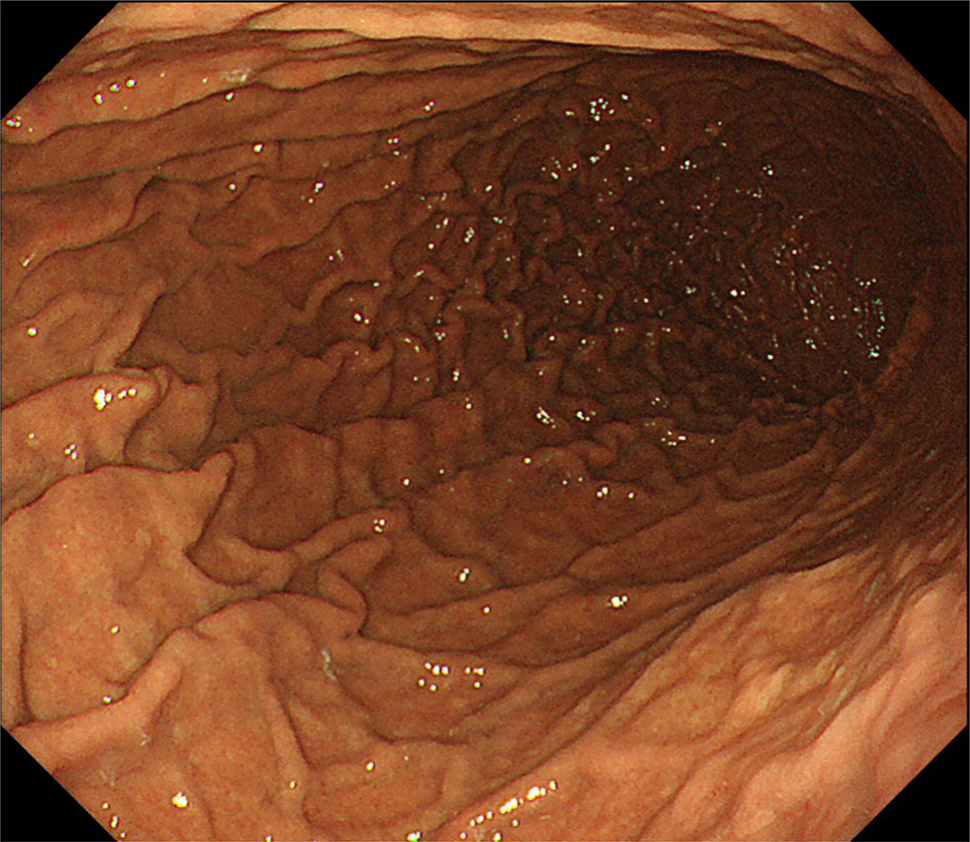
Esophagogastroduodenoscopic findings 1 year after discharge. Gastric polyps were not observed and the vulnerability of gastric mucosa had improved

He was brought to our hospital because he was diagnosed with anemia (hemoglobin [Hb] 8.0 g/dL) during a medical checkup. During EGD, whitish, edematous multiple polyps in the antrum and corpus of the stomach without hemorrhages were noted, which made us suspect FGPs according to endoscopic findings, and a biopsy was subsequently performed (Fig. 1a). Pathological findings revealed dilated and proliferated fundic glands with foveolar hyperplasia. A colon endoscopy did not reveal any abnormalities. However, the patient complained of general fatigue and a large amount of tarry stools was observed. The patient was admitted to our hospital 8 days after his initial EGD. The patient’s family history was unremarkable and he did not have a history of allergies. His alcohol consumption was around 350 mL/day of beer for 20 years. A blood test showed iron deficiency anemia. Serum anti-*H. pylori* antibody level and antigen in stool were within the normal range. The anti-gastric parietal cell antibody test was also negative (Table 1). Computed tomography did not identify any cause for gastrointestinal bleeding. The patient underwent a capsule endoscopy, which did not reveal any abnormalities in the small intestine, although a large amount of black residue was observed in the stomach. EGD revealed multiple polyps and mild oozing was observed from the polyps and gastric mucosa (Fig. 1b). We tried to perform endoscopic hemostasis by using hemostatic forceps in the soft coagulation mode, but the observed hemorrhaging increased. Because the gastric mucosa was vulnerable to further hemorrhage and the oozing still persisted, we resected three bleeding polyps. The patient progressed satisfactorily and he was temporarily discharged. Two weeks after discharge, the patient was rehospitalized with tarry stools. EGD revealed coagula in the stomach, and multiple polyps in the corpus were observed to be hemorrhagic (Fig. 1c, d). Endoscopic mucosal resection and polypectomy were performed at 21 sites. Furthermore, we stopped the administration of a PPI. Microscopically, characteristics of 20 polyps in the body of the stomach were almost same. Significant cystic dilatation of glands was observed. Polyps showed characteristics of hyperplasia of the foveolar epithelium, extended fundic glands and edema of the stroma, suggesting lesions that differed from typical FGPs (Fig. 2-1). Together with hyperplasia of the foveolar epithelium and extended mucous glands, the proliferation of parietal and chief cells were also observed, but not parietal cell protrusion or inflammatory cell infiltration. Apoptotic bodies were detected in the boundary region between fundic and neck mucous glands (Fig. 2-2). Crypt epithelial cells (MUC5AC) were mainly observed superficially and cervical mucous cells (MUC6) were observed beneath the crypt epithelium. Fundic glands were positive for H+/K+-ATPase, and showed a proliferation of predominantly parietal cells. Only a very slight accumulation of chromogranin A staining was found, and enterochromaffin-like (ECL) cells were not detected. Few positive cells were found by Ki67 staining and polyps lacked dysplasia (Fig. 2-3). Aquaporin-4 (AQP4) and KCNQ1-positive parietal cells and dilated mucous glands were found from the basal side to the apical side of the mucosa. The extension of the distribution of AQP4 and KCNQ1-positive cells toward the apical side of the fundic glands was observed (Fig. 2-4). Overexpression of gastrin receptors was not detected. A reddish polyp in the antrum showed infiltration of inflammatory cells and it was similarpathologically to inflammatory polyp. Gastrin overexpression was not observed in the antral polyp immunohistochemically (Fig. 2-4).

**Table 1:** Laboratory data on admission

Hematology	
WBC	6300 /μL
RBC	413 × 10^4^ /μL
Hb	7.6 g/dL
Ht	27.3 %
Plt	25.6 × 10^4^ /μL
Coagulation	
PT	76.0 %
APTT	37.6 s
Blood chemistry	
TP	7.2 g/dL
Alb	4.6 g/dL
T-Bil	0.35 g/dL
AST	14 IU/L
ALT	13 IU/L
ALP	174 IU/L
LDH	179 IU/L
γ-GTP	31 IU/L
ChE	324 U/L
BUN	10 mg/dL
Cre	0.81 mg/dL
Na	143 mmol/L
K	3.8 mmol/L
Cl	105 mmol/L
Glu	103 mg/dL
Fe	19 μg/dL
TIBC	551 μg/dL
Ferritin	4 ng/dL
HbA1c	5.6 %
Gastrin	89 pg/mL
Tumor marker	
CEA	2.0 ng/mL
CA19-9	11 U/mL
Serological test	
CRP	0.0 mg/dL
HBsAg	(–) U/mL
HCVAb	(–)
Hp-IgG	<3 U/mL
Anti-parietal cell antibody	(–)
Hp antigen in stool	(–)

The patient was discharged under continued selective serotonin reuptake inhibitors (SSRIs) administration and has been making satisfactory progress in the outpatient department ever since. EGD showed few gastric polyps 1 month after discharge. The serum gastrin level after PPI withdrawal maintained within the normal range (120 pg/mL). The vulnerability of gastric mucosa had improved and gastric polyps were not observed 1 year after discharge (Fig. 3).

## Discussion

We report herein a case of hemorrhagic gastric polyps, which were pathologically characterized by both hyperplastic and FGPs during long-term PPI administration, although they showed a morphology similar to FGPs as determined endoscopically. Of the polyps generally found during long-term PPI administration, FGPs are the most common [1, 2]. According to previous research in Japan [3], FGPs were found in 13.6%, and hyperplastic polyps in 8.9% of patients during long-term PPI administration.

However, one group reported that FGPs were not induced by PPI therapy [4]. Although the mechanism whereby PPI administration causes FGPs is unknown in detail, a relationship with *Helicobacter pylori* negative patients has been reported [5]. Tsuchigame et al. reported that FGPs were present in the region blackened by congo red spray and it was considered that FGPs were observed in stomachs with less atrophy [6]. In the present case, a patient who received long–term PPI administration, *H. pylori* was negative and there was no gastric atrophy, therefore *H. pylori* negative may be associated with the FGP-like lesion. Elevated blood gastrin levels are also assumed to cause hyperplastic polyps. Gastrin has a mucosal proliferative effect that enhances the effects of growth factors, such as those of the epidermal growth factor (EGF) and tumor growth factor-alpha (TGF-α) families, and promotes the growth of crypt epithelial cells [7]. In the present case, a significant decrease in multiple gastric polyps was observed immediately after PPI withdrawal 1 month after discharge, which highlights strongly how PPIs are suspected to cause gastric polyps. PPI administration for a year or more increased the risk of occurrence of polyps significantly, which further increased as the administration term lengthened [8].

The histopathological changes most commonly observed during PPI administration were parietal cell protrusion/parietal cell hyperplasia, edema of stroma and cystic dilation of the fundic gland duct [9]. In this case, hyperplasia of parietal cells was not found, corresponding with the results of a previous report [9]. Bleeding from multiple FGPs was suspected from endoscopic examination, but pathological tests revealed that polyps consisted of the mixed hyperplasia of foveolar epithelium, and cystic dilatation of fundic glands. Edema of the stroma and extended fundic glands was also observed. Recently some investigators reported the association between acid suppression by PPI and gastric polyps with water channel aquaporin-4 and potassium channel KCNQ1 expression. We performed the immunohistochemical examination of aquaporin-4 (AQP4) and KCNQ1. AQP4 and KCNQ1-positive parietal cells, and dilated mucous glands were found broadly from the basal side to the apical side of the mucosa. The extension of the distribution of AQP4 and KCNQ1-positive cells toward the surface of the fundic glands was observed according to the previous report [10, 11]. These findings differed from the usual FGPs, and were thought to be lesions associated with long-term PPI administration. The serum gastrin level was not increased from that measured upon admission and also immunohistochemical expression of gastrin and gastrin receptors on these polyps was not increased, which suggests that polyp development or growth was not associated with a gastrin-dependent pathway. Lee reported the importance of apoptosis in the histopathology of drug related lesions [12]. In this case, a patient who received long–term PPI administration, apoptotic bodies were detected between fundic and neck mucous glands. Therefore, the appearance of apoptotic bodies suggests drug related mucosal change and pathogenesis of the FGP-like lesion.

This case was characterized by the relative difficulty of treatment because of hemorrhagic activity. In this case, re-bleeding occurred many times after polyp resections, and no clear reasons for the observed hemorrhagic activity have been identified as yet. However, polyp resections combined with hyperplastic components may have led to such hemorrhagic activity. It has been suggested that with regard to hemorrhaging, the long-term administration of PPI should be discontinued. In recent years, a relationship between SSRIs and gastrointestinal bleeding has been found: it is thought that platelet aggregation can be reduced by decreasing the serotonin level of platelets [13]. However, our department previously found that SSRIs did not increase the risk of upper gastrointestinal mucosal damage and gastric polyps as determined endoscopically [14]. Since the development of FGPs and hyperplastic polyps due to the long-term SSRI administration has not been reported, the cause of bleeding in this case may be a reduction of platelet aggregation due to SSRI administration. As for the causes of hemorrhagic gastric mucosa and polyps, we suggest that hyperplasia of foveolar glands may lead to membrane vulnerability and that long-term SSRI administration is involved in hemorrhagic mucosa.

In summary, we experienced a case in which bleeding from polyps in the form of fundic glands during the long-term administration of PPIs was treated by endoscopic resection. Based on the case presented here, it is rare to bleed from polyps that resemble FGPs, but it may be that the long-term administration of PPIs may induce such bleeding. Further pathological investigations will be needed on polyps during long-term PPI administration.
